# Comparison Between Conventional Intervention and Non-immersive Virtual Reality in the Rehabilitation of Individuals in an Inpatient Unit for the Treatment of COVID-19: A Study Protocol for a Randomized Controlled Crossover Trial

**DOI:** 10.3389/fpsyg.2021.622618

**Published:** 2021-02-24

**Authors:** Talita Dias da Silva, Patricia Mattos de Oliveira, Josiane Borges Dionizio, Andreia Paiva de Santana, Shayan Bahadori, Eduardo Dati Dias, Cinthia Mucci Ribeiro, Renata de Andrade Gomes, Marcelo Ferreira, Celso Ferreira, Íbis Ariana Peña de Moraes, Deise Mara Mota Silva, Viviani Barnabé, Luciano Vieira de Araújo, Heloísa Baccaro Rossetti Santana, Carlos Bandeira de Mello Monteiro

**Affiliations:** ^1^Departamento de Medicina (Cardiologia), Escola Paulista de Medicina da Universidade Federal de São Paulo (UNIFESP), São Paulo, Brazil; ^2^Grupo de Pesquisa e Aplicações Tecnológicas em Reabilitação da Escola de Artes, Ciências e Humanidades da Universidade de São Paulo (PATER EACH USP), São Paulo, Brazil; ^3^Faculdade de Medicina da Universidade Cidade de São Paulo (UNICID), São Paulo, Brazil; ^4^Serviço de Fisioterapia do Hospital São Paulo, Universidade Federal de São Paulo (HSP UNIFESP), São Paulo, Brazil; ^5^Executive Business Centre, Orthopaedic Research Institute, Bournemouth University, Bournemouth, United Kingdom

**Keywords:** coronavirus, telerehabilitation, virtual reality exposure therapy, hospitals, rehabilitation, autonomic nervous system, physical functional performance

## Abstract

**Background:** The new human coronavirus that leads to COVID-19 (coronavirus disease 2019) has spread rapidly around the world and has a high degree of lethality. In more severe cases, patients remain hospitalized for several days under treatment of the health team. Thus, it is important to develop and use technologies with the aim to strengthen conventional therapy by encouraging movement, physical activity, and improving cardiorespiratory fitness for patients. In this sense, therapies for exposure to virtual reality (VR) are promising and have been shown to be an adequate and equivalent alternative to conventional exercise programs.

**Aim:** This is a study protocol with the aim of comparing the conventional physical therapy intervention with the use of a non-immersive VR software during COVID-19 hospitalization.

**Methods:** Fifty patients hospitalized with confirmed diagnosis of COVID-19 will be divided in two groups under physiotherapy treatment using conventional or VR intervention: Group A: participants with COVID-19 will start the first day of the protocol with VR tasks in the morning and then in the second period, in the afternoon, will perform the conventional exercises (*n* = 25) and Group B: participants with COVID-19 will start the first day with conventional exercises in the morning and in the second period, in the afternoon, will perform activity with VR (*n* = 25). All participants will be evaluated with different motor and physiologic scales before and after the treatment to measure improvements.

**Conclusion:** Considering the importance of benefits from physical activity during hospitalization, VR software shows promise as a potential mechanism for improving physical activity. The results of this study may provide new insights into hospital rehabilitation.

**Trial Registration:** ClinicalTrials.gov, identifier: NCT04537858. Registered on 01 September 2020.

## Introduction

At the end of 2019, the world saw the emergence of a new human coronavirus that was spreading rapidly and with a high degree of lethality (Huang et al., 2020; Singhal, 2020). The virus was called SARS-CoV-2 (severe acute respiratory syndrome coronavirus 2), responsible for causing COVID-19 (coronavirus disease 2019) (Baig et al., 2020; Chen et al., 2020; Weston and Frieman, 2020; Zhou et al., 2020).

The majority of people who acquired the virus did not show any significant symptoms (Chen et al., 2020), whereas 15% of people who had the virus developed mild or moderate symptoms, the most common of which were fever (85–90%), cough (65–70%), fatigue (35–40%), dyspnea (15–20%), and myalgia (10–15%) (Guan et al., 2020; Huang et al., 2020; Madabhavi et al., 2020).

Different from existing viruses that cause SARS and Middle East respiratory syndrome (De Wit et al., 2016; Madjid et al., 2020; Paules et al., 2020), COVID-19 symptoms cause early complications such as acute respiratory distress syndrome sepsis and multiple organ failure requiring admission to the intensive care unit (Yang et al., 2020), which might lead to overload of care services in public and private sectors (Sheehy, 2020; Wu and McGoogan, 2020). In addition to the high rate of hospitalization, a major difference from COVID-19 is the average hospitalization period, which is usually 12 days (Guan et al., 2020) in an outpatient setting, and if there is a need for intensive care, this period can be extended to ~22 days (Yang et al., 2020; Zhou et al., 2020).

Considering the need for hospitalization, the rehabilitation protocol includes multidisciplinary teams, with physiotherapy treatment being recognized as an important intervention. According to Lew et al. (2020), physiotherapy in the treatment of COVID-19 consists mainly of physical care where the patient may have low physical fitness, shortness of breath after exertion, and muscle atrophy (including respiratory muscles, trunk, and limb muscles). It is important to emphasize that hospitalized patients receive physiotherapy treatment at the bedside once a day for those in moderate need, and three times a day for those in high need (Smith et al., 2020). This intervention uses progressive exercises that can be selected so that patients can gradually recover the level of activity observed before the onset of the disease (Zhao et al., 2020).

Thus, innovations using virtual reality (VR) during intervention in outpatient rehabilitation services are important to minimize functional deficits that might otherwise lead to permanent disability (Boldrini et al., 2020; Coraci et al., 2020). Where other VR systems are cost-prohibitive, low-cost VR promotes an important cost-effectiveness to public health service, providing an effective tool to health workers to work as adjunctive to routine with inpatients with less effort in patient handling (Zanaboni et al., 2016; Ford et al., 2018; Shields et al., 2018). Therefore, the use of computational tasks in a virtual environment can be an interesting option (de Freitas et al., 2019; de Moraes et al., 2020; Leal et al., 2020; Stam et al., 2020).

The use of tasks in virtual environments has grown a lot in recent years, showing itself as an adequate alternative and equivalent to conventional exercise programs (Bond et al., 2019; García-Bravo et al., 2019), and the benefits of physical exercise associated with VR have shown a promising impact in improving the patient's self-efficacy for physical training, in addition to providing an engaging environment for the patient (Dias et al., 2019). Moreover, the VR can enable the rehabilitation team to control different variables such as speed, task size, and more important creative tasks to promote motivation (Fernani et al., 2017; Prado et al., 2017; da Silva et al., 2020b).

In addition to improve motor performance and functionality, it has been shown to increase motivation and consequent engagement to rehabilitation. In a recent systematic review of VR and video games for cardiac rehabilitation programs, all publications pointed to improvement of the motivation and engagement to therapy as the main advantage derived from the use of VR rehabilitation; the subjects reported that it was a fun and interactive form of treatment (García-Bravo et al., 2019).

Despite positive existing studies with the use of VR in different respiratory disabilities, such as chronic obstructive pulmonary disease (Mazzoleni et al., 2014; Jung et al., 2020; Rutkowski et al., 2020), exercises training on thoracic hyperkyphosis, and respiratory parameters in young women (Taslimipour et al., 2020), research in a hospital environment is still scarce, and more studies are needed to verify benefits and possible applications.

According to Bond et al. (2019), the requirement of physical exercise inherent to a video game's activities (exergames) is shown to have a promising impact in improving patient self-efficacy for exercise training using digital hardware (e.g., the Nintendo Wii® or the Xbox Kinect®), once interactive VR promotes increased heart rate (HR), rating of perceived exertion (RPE), and physical activity (Bond et al., 2019; García-Bravo et al., 2019). Therefore, patients using exergames rather than conventional exercise routines (e.g., walking, running, or cycling) have been reported to exercise for longer periods, meeting moderate to vigorous physical activity levels, with the perception of “work” being lower or less intense, and more enjoyable, contributing to healthier lifestyle initiatives (Glen et al., 2017; Gomez et al., 2018; Polechoński et al., 2019).

Considering the above deliberations, we have organized a randomized controlled crossover protocol to investigate rehabilitation of patients hospitalized with COVID-19. The aim of this protocol is to find out whether the motor performance, functionality, engagement, motivation, and physiological parameters are different during conventional or virtual intervention. We are interested in comparing conventional cardiovascular and musculoskeletal physical therapy interventions with typical movements and mobilization in different postures such as sitting and standing, using VR software that provides a game in which the participants continually move upper limbs and trunk to finish a motor task proposed by the application. Participants will therefore alternate their rehabilitation intervention (conventional or virtual) during their hospitalization period. Furthermore, we will be using a satisfaction scale to verify the level of engagement in the therapy during the rehabilitation program.

We hypothesize that all participants will show improvement in motor performance, functionality, and physiological parameters independent of the intervention (conventional or virtual environment); however, the use of a virtual task will provide more engagement and motivation. If our hypothesis is proven, it might be the beginning of a technological innovation in cardiorespiratory rehabilitation in hospitals.

## Methods/Design

We registered this trial on ClinicalTrials.gov (NCT04537858). This article has been reported in accordance with the Standard Protocol Items Recommendations for Interventional Trials (SPIRIT) (Chan et al., 2013a,b; Tables 1, 2).

**Table 1:** SPIRIT: description of the study protocol, schedule of enrolment, interventions, and assessments.

**Study period**												
	**Enrolment**	**Allocation**	**Post-allocation**								**Close-out**	
**Timepoint**	**0**	**0**	**t**_**1**_	**t**_**2**_	**t**_**3**_	**t4**	**t5**	**t6**	**t7**	**t8**	**t9**	**t10**
			**1 day**	**2 days**	**3 days**	**4 days**	**5 days**	**6 days**	**7 days**	**8 days**	**7 days after discharge**	**1 month after discharge**
**ENROLLMENT**												
Eligibility screen	X											
Informed consent	X											
Assessment scales	X											
Allocation		X										
**INTERVENTIONS**												
Group A			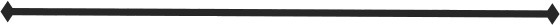									
Group B			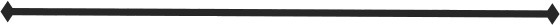									
**ASSESSMENT SCALES**												
Barthel Index	X									X	X	X
Timed Up and Go (TUG)			X							X	X	X
Medical Research Council (MRC)			X							X	X	X
Brunel Mood Scale (BRUMS)			X							X	X	X
Enjoyment Scale (ES)			X	X	X	X	X	X	X	X	X	X
Visual Analogical Satisfaction Scale (VASS)			X	X	X	X	X	X	X	X	X	X
**PHYSIOLOGICAL ASSESSMENT**												
Heart rate variability (HRV)			X							X	X	X
The Borg Rating of Perceived Exertion (Borg scale)			X	X	X	X	X	X	X	X	X	X
Oxygen saturation (Sao_2_)			X	X	X	X	X	X	X	X	X	X
Respiratory rate (RR)			X	X	X	X	X	X	X	X	X	X
Heart rate (HR)			X	X	X	X	X	X	X	X	X	X

**Table 2:** Trial characteristics based on WHO Trial Registration Data Set.

**Data category**	**Trial information**
Primary registry and trial identifying number	ClinicalTrials.gov, ID: NCT04537858
Date of registration in primary registry	01 September 2020 on
Secondary identifying numbers	Ethics Committee of the Federal University of São Paulo, under the number CAAE: 33244620.5.0000.5505
Source(s) of monetary or material support	Coordenação de Aperfeiçoamento de Pessoal de Nível Superior–Brasil
Primary sponsor	University of São Paulo
Secondary sponsor(s)	NA
Contact for public queries	TDS, CBMM
Contact for scientific queries	TDS, CBMM
Public title	Conventional intervention and non-immersive virtual reality in COVID-19
Scientific title	Comparison Between Benefits of Conventional Intervention and Non-immersive Virtual Reality in the Rehabilitation of Individuals in an Inpatient Unit for the Treatment of COVID-19: A Study Protocol
Country of recruitment	Brazil
Health condition(s) or problem(s) studied	COVID-19
Interventions	Group A: Subjects will start the first day of the protocol with virtual reality intervention and then in the second period will perform the conventional intervention. Group B: Subjects will start the first day with conventional intervention and in the second period will perform activity with virtual reality intervention. Both therapies will be performed on the same day at different times for 10 consecutive days
Key inclusion and exclusion criteria	Inclusion criteria: Agreement to participate in the research from themselves by signing consent form, a clinical and posterior blood analysis of COVID 19 infection, adults with age ranging from x to xx years. Exclusion criteria: Do not understand the tasks; the understanding of the task will be evaluated through five attempts at each task in virtual reality; do not want to use virtual reality task; was not in a physical condition to practice a functional task; use of an intravenous device that makes it impossible to move the arms; cardiac arrhythmias and atrioventricular block; congenital anomalies, such as congenital heart defects, pulmonary malformations; and patients who use drugs that interfere with ANS, such as antiarrhythmic agents
Study type Interventional allocation	Randomized
Masking	Randomization and data analyst
Assignment	Crossover
Primary purpose	Treatment
Date of first enrolment	June 2020
Target sample size	50
Recruitment status	Recruiting
Primary outcome(s)	Functional capacity improvement
Key secondary outcome(s)	HRV improvement

### Overview of the Study Design

A randomized controlled crossover protocol will be conducted, and all participants will undertake interventions in non-immersive VR–virtual intervention (with a serious game in web platform) and conventional intervention (with cardiovascular and musculoskeletal conventional physical therapy). All participants will be randomly divided in two groups: Group A will start the first day of the protocol with virtual intervention in the morning (their first rehabilitation session of the day) and the conventional intervention in the afternoon (their second rehabilitation session of the day). Group B will perform the reverse protocol (i.e., starting the first day of the protocol with conventional intervention in the morning and the virtual intervention in the afternoon) in a crossover design, during the hospitalization period (varying from 2 to 8 days according to individual needs). We chose two interventions per day, as it is the protocol of the Hospital of the Federal University of São Paulo (Hospital São Paulo) for inpatients in ambulatory unit.

### Recruitment

Fifty participants will be recruited through the Hospital of the Federal University of São Paulo (Hospital São Paulo) located in São Paulo State, Brazil. Those interested in participation will undergo a detailed screening using the eligibility criteria for enrolment in the study. The sample size was calculated using a statistical software (G^*^Power 3.1.5) on the main outcome measure (i.e., the motor score). This calculation was based on data from five patients (pilot study). The power was 0.80; the α was 0.05, and the effect size was 0.65 (Cohen *d*). The sample estimation indicated that 40 participants would be necessary (i.e., 20 per group), and with an adjustment to allow for a withdrawal rate (20%), we will recruit 50 participants.

### Inclusion Criteria

Participants will be included in the study if (1) they are aged between 18 and 90 years; (2) they are diagnosed as having COVID-19 by reverse transcription–polymerase chain reaction, immunoglobulin M, and immunoglobulin G (Corman et al., 2020; Zhong et al., 2020); (3) they are undergoing physical therapy during their hospitalization; and (4) they are able to sign consent form.

### Exclusion Criteria

Participants will be excluded if (1) they do not understand the tasks—the understanding of the task will be evaluated through five attempts at each task in VR; (2) they do not want to use VR; (3) they are not in a physical condition to practice a motor task; (4) the use of an intravenous device makes it impossible to move the arms; (5) they have cardiac arrhythmias and an atrioventricular block or cardiac pacemaker; (6) they have congenital anomalies such as congenital heart defects or pulmonary malformations; and (7) they use drugs that interfere with autonomic nervous systems (ANSs), such as antiarrhythmic agents.

### Withdrawal Criteria

Participants will be withdrawn from the study if they are not willing to continue.

### Randomization

Participants will be randomly allocated to either Group A or Group B with a 1:1 allocation defined by a website (randomization.com). As we will have the participant's characteristics, immediately after the randomization the age and functional capacity (Barthel Index) will be compared between groups. If the groups are not homogeneous, a new randomization will be carried out. This protocol will be repeated until there is no difference between age and Barthel Index among groups (in a maximum of first three attempts at randomization, we always have homogeneous groups). Randomization will be under the control of a blinded investigator who will be the only person allowed to manage the electronic security file of the randomization to locate the individuals. The investigator applying the interventions will then be informed about which group the participant was allocated to so they can conduct the protocol in the correct order of interventions.

### Assessment Scales

We will use functional capacity, muscle strength, mood, enjoyment, satisfaction scales, and physiological assessment, which will be presented as follows:

#### Motor Function

For functional capacity, the following tests will be used: Barthel Index, Timed Up and Go (TUG), and Medical Research Council (MRC).

##### Barthel Index

The Barthel Index is a 10-item instrument measuring functional independence in personal activities of daily living—it reaches a score of 100. The Barthel Index is quick and easy to complete. The scoring instructions used for the Barthel Index have been modified to make the contribution of cognitive problems to functional dependency more explicit. The topics evaluated are as follows: (1) feeding, (2) moving from wheelchair to bed and return, (3) personal toileting (grooming), (4) getting on and off toilet, (5) bathing self, (6) walking on a level surface, (6a) propelling a wheelchair, (7) ascending or descending stairs, (8) dressing and undressing, (9) controlling bowels, and (10) controlling bladder. The Barthel Index has been shown to be appropriate for the assessment of patients' changes over time (Novak et al., 1996; Houlden et al., 2006; Eichhorn-Kissel et al., 2011).

##### Timed Up and Go

The TUG test is a reliable, cost-effective, safe, and time-efficient way to evaluate overall functional mobility. The TUG may be used to assess and monitor physical activity in younger adults, especially those with physical and mental health risk factors. A sturdy armchair with a back will be placed at the end of a hallway or an area with space to perform the test. A piece of tape will be placed on the floor 3 m away from the front edge of the chair. Patients will be seated in the chair with back against the chair back, arms resting on the armrests, and given instructions on how to complete the task, including walking at a normal (rather than rapid) speed. The TUG will require patients to stand up out of the chair, walk 3 m, turn around, walk back to the chair, and sit down. Patients will be given the following instructions: “Stand up on the word ‘go,' walk to the tape, turn around, walk back to the chair, and sit down.” The timing of the test will begin at the word “go” and end when the participant is seated. Patients will perform the test one time; if a clear error is made, they will be asked to repeat the TUG. The dependent variable will be the test execution time (Bohannon, 2006; Herman et al., 2011; Kear et al., 2017).

##### Medical Research Council

This is an instrument adapted to assess muscle strength in critically ill patients. The result is obtained through the evaluation of six movements of upper limbs and lower limbs, and the strength is graded between 0 (plegia) to 5 points (normal strength). The maximum score is 60 points; values of <48 may indicate that the patient has muscle weakness (Craig et al., 2008; Schefold et al., 2020).

#### Mood, Enjoyment, and Satisfaction Scales

For mood, enjoyment, and satisfaction, the following tests will be used: Brunel Mood Scale (BRUMS), Enjoyment Scale (ES), and Visual Analogical Satisfaction Scale (VASS).

##### Brunel Mood Scale

This scale was developed to allow a quick measurement of the mood of adults and adolescents. BRUMS contains 24 simple mood indicators, such as feelings of anger, disposition, nervousness, and dissatisfaction that are noticeable by the individual being assessed. The evaluated responds to the scale according to how they feel about such sensations. The score is 5 points (0 = nothing to 4 = extreme). The question format is “How do you feel now,” although other forms: “How have you felt this past week, including today” or “How do you normally feel” can be used. BRUMS takes about 1 to 2 min to complete. The 24 indicators of the scale comprise six subscales: anger, confusion, depression, fatigue, tension, and vigor (Rohlfs et al., 2008; Sties et al., 2014).

##### Enjoyment Scale

An enjoyment scale using smiley faces (0 is “not fun at all,” 1 is “boring,” 2 is “a bit of fun;” 3 is “fun,” and 4 is “great fun”) will be applied after the end of the game sequences, as the motivation may be related to the motor proficiency level.

This scale was developed by Jelsma et al. (2014) to evaluate how people feel when interacting with proposed non-immersive VR games. It was used in other studies using different games (Farhat et al., 2016; Smits-Engelsman et al., 2017) and by da Silva et al. (2020a) with the same game used in this study. In the present study, the scale will be applied in the first and last days of the protocol to verify the participant's level of satisfaction with the games presented.

##### Visual Analogical Satisfaction Scale

The 10-cm VASS assesses the level of satisfaction of the interviewed individuals. Patients will answer the questionnaire and be asked to mark a vertical line on the scale indicating satisfaction with rehabilitation, where zero (0) indicates very dissatisfied, and 10 indicates very satisfied (Brokelman et al., 2012; Voutilainen et al., 2016).

#### Physiological Assessments

For physiological assessment, the following tests will be used: The Borg Rating of Perceived Exertion (Borg Scale), heart rate variability (HRV), oxygen saturation (Spo_2_), respiratory, and HR.

##### The Borg Rating of Perceived Exertion

The Borg Scale is a tool for monitoring the intensity of physical effort; it is one of the most useful instruments for the evaluation and quantification of the sensations of physical effort, also known as RPE. This is used both in the areas of high-performance sports and physical rehabilitation to monitor the changes caused by physical exercise in the cardiorespiratory, metabolic, and neuromuscular systems (Zamunér et al., 2011; Williams, 2017).

##### Heart Rate Variability

We will use HRV to analyze ANSs before, during, and after the intervention for recovery assessment. The analysis will follow the guidelines of the (Heart rate variability: standards of measurement, physiological interpretation and clinical use. Task force of the European Society of Cardiology and the North American Society of pacing and electrophysiology, 1996). The strap (for data collection) will be positioned on the participant's chest, and the Polar V800 (Polar Electro, Finland) HR receiver will be positioned next to it. HRV will be recorded after the initial assessments at rest for 20 min. For analysis of HRV data at rest, 1,000 consecutive resting rate intervals will be used (da Silva et al., 2018; Moraes et al., 2019). Heart rate will be recorded beat by beat throughout the protocol by the Polar V800 HR receiver, and resting rate intervals recorded by the monitor will be transferred to the Polar ProTrainer program, which allows HRV visualization and cardiac period extraction in the “txt.” file format.

Moderate digital filtering will be performed in the program itself. HRV analysis will be performed using linear (time and frequency domain) that will be analyzed using Kubios HRV® software (Kubios HRV v.1.1 for Windows, Biomedical Signal Analysis Group, Department of Applied Physics, University of Kuopio, Finland) and non-linear methods (Vanderlei et al., 2009).

##### Oxygen Saturation

Pulse oximetry is widely used for patients who need continuous monitoring of Spo_2_. Its main purpose is the early detection of hypoxemia in various situations and the monitoring of perfusion and circulation; monitoring is non-invasive (Diccini et al., 2011). Before starting therapy with VR and conventional therapy, Spo_2_ will be measured. At the end of the respective therapies, Spo_2_ will be checked again.

##### Respiratory Rate and Heart Rate

Respiratory rate (RR) and HR will be measured before starting and at the end of both VR and conventional interventions.

### Blinding

The statistical analyst will be blinded throughout the treatment; i.e., the statistician will only know that there is a Group A and Group B carrying out the evaluations without information of what the treatment is, so that treatment of data is impartial.

### Assessment Protocol

The assessment protocol will have the following sequence. The functional scale (Barthel Index) will be undertaken first to ensure balance in randomization as this is a key variable. This is followed by group randomization process. After that, assessment scales will be undertaken in a separate room (BRUMS, MRC, and TUG) and one physiological assessment (HRV). The assessment part of the protocol will take around 1 h.

Borg Scale, Spo_2_, RR, and HR will be evaluated before and after each intervention. At the end of each day, ES and VASS questionnaires will be used to determine the individual's final perception of the intervention.

### Intervention

After performing all the tests and questionnaires of the initial evaluation, the individuals will be divided into two groups: Group A: participants with COVID-19 who will start the first day of the protocol with virtual intervention and then in the second period will perform the conventional intervention (*n* = 25); and Group B: participants with COVID-19 who will start the first day with conventional intervention and in the second period will perform activity with VR intervention (*n* = 25). After the application of therapies, final evaluations will be carried out. The rehabilitation protocol will be applied during hospitalization, for 2–8 consecutive days, unless they withdraw from the study—the number of days may vary due to length of hospitalization.

Participants will perform the tasks individually in the hospitalization sector, in the presence of the evaluator responsible for providing the intervention and recording the results, considering the patient's general condition. According to the recommendations of the European Respiratory Society, conventional physiotherapy includes (1) mobilization: referring to physical activity sufficient to elicit acute physiological effects that enhance ventilation, central and peripheral perfusion, circulation, muscle metabolism, and alertness and are countermeasures for venous stasis and deep vein thrombosis; and (2) respiratory therapy: to improve global and/or regional ventilation and lung compliance and to reduce airway resistance (Gosselink et al., 2008).

### Conventional Intervention

We stipulate a protocol that considers motor physiotherapy conducted for 10 min, which will be performed at the bedside with mobilization exercises to activate the upper and lower limbs, orthostatic training, static and dynamic balance, and walking through the corridor.

### Virtual Intervention

During the protocol, the participants will perform tasks in a non-immersive VR environment for 10 min. Thus, we will use the MoveHero game (for details and publication, see Martins et al., 2019) that provides mobilization exercises to activate the upper and lower limbs, orthostatic training, and static and dynamic balance. The software that will be used was developed by the Research Group and Technological Applications in Rehabilitation group from the School of Arts, Sciences, and Humanities of the University of São Paulo and can be accessed at www.movehero.com.br/en.

#### MoveHero

**Figure 1:**
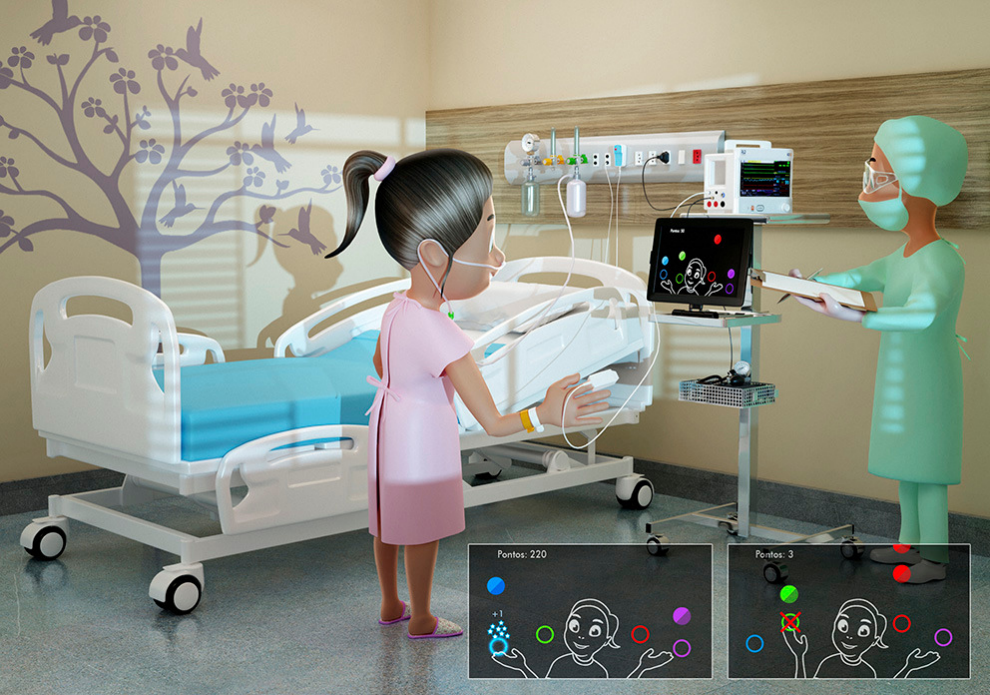
Representative design of the MoveHero software performed during treatment intervention, with representation of hits (bottom left figure) with sphere turning into blue with stars, and misses (bottom right figure) with a red X.

MoveHero, as presented by Martins et al. (2019), is a game that displays falling spheres in four imaginary columns on the computer screen, with a musical rhythm selected by the researcher. This is also considered a coincident timing task; the action is to react (using the upper limbs) and not let the balls pass the fixed targets. The spheres should only be intercepted when they reach the targets allocated in parallel (at two height levels), two on the left (left position targets A and B) and two on the right of the participant (right position targets C and D), as shown in Figure 1. The virtual contact is performed by the avatar of the individual, i.e., a representation of the individual appears on the computer screen. The individuals move their arms and trunk (only if they can move the trunk) in front of the webcam to coincide with the moment the ball touches the target. The individual is positioned at a distance of ~1.5 m from the computer monitor and waits for the balls (which fall randomly on each target) to drop. The avatar's hand should reach the target sphere along with the arrival of the ball, and the game offers feedback on correctness and error by means of changing the spheres' color (green for correct and a red line for error).

Thus, the participants will play the MoveHero game in a bedside standing or seated position (depending on the patient's capacity), where they have to move their arms and body/trunk to catch the falling spheres. After this motor intervention, the participants will also receive respiratory physiotherapy for 10 min, during which they will perform exercises of respiratory reeducation.

### Procedure

**Figure 2:**
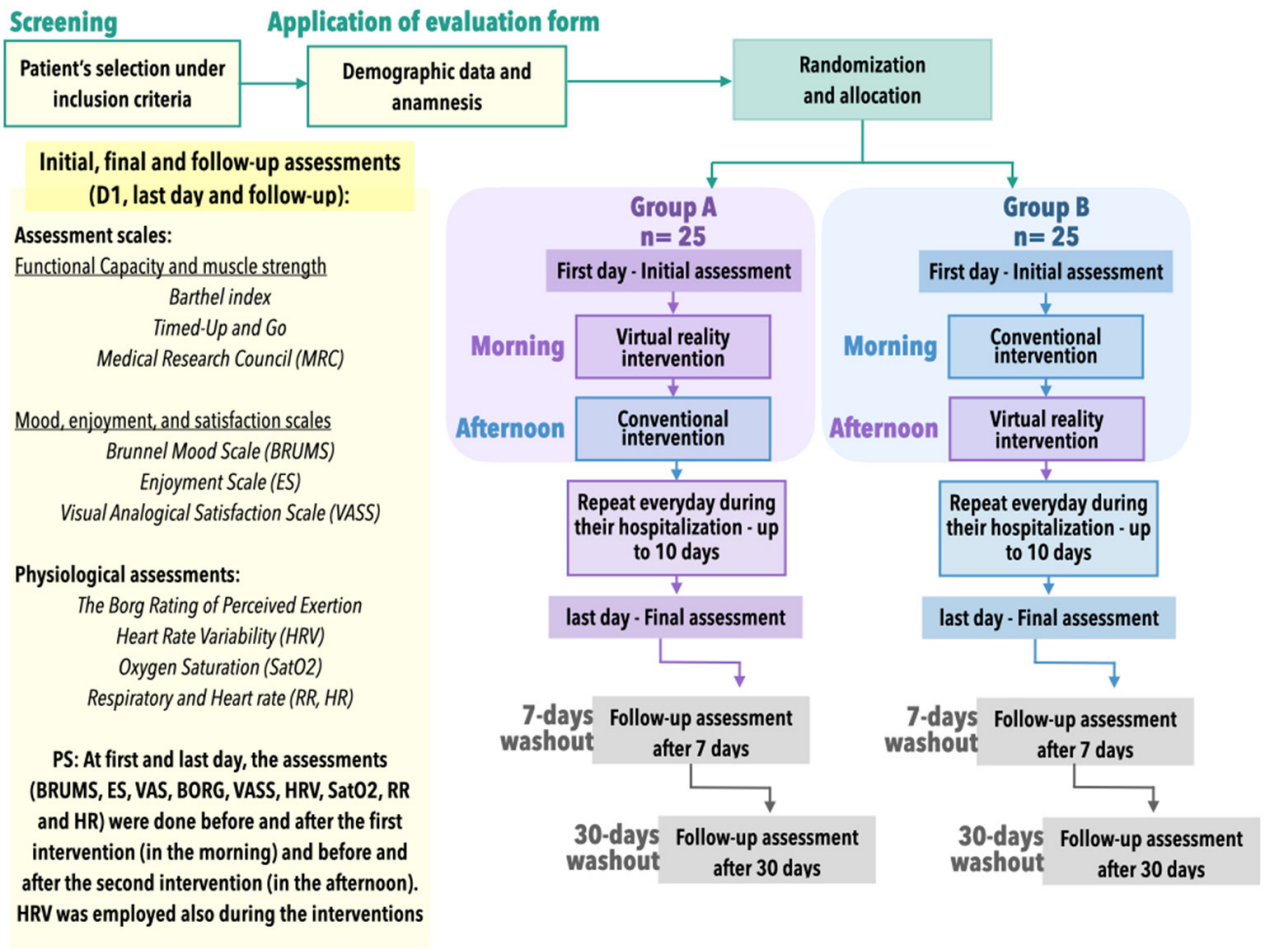
Study design.

Immediately before the beginning of each therapy (virtual intervention or conventional intervention), two assessment scales will be made (BORG Scale, Visual Analog Scale) and four physiological assessments [HRV, Spo_2_, RR, and HR]. Group A will start the first day of the protocol with virtual intervention (in the morning) and then in the second period (in the afternoon) will perform the conventional intervention, and Group B will perform the reverse protocol, in a crossover format. It will be applied for up to 8 consecutive days. On the last day, assessment scales will be repeated in a separate room, as well as the physiological assessments. After 7 days and 1 month of hospital discharge, all assessment scales will be undertaken at patients' homes, and the virtual intervention will be applied again (Figure 2).

### Primary Outcome

We will observe changes in the ANSs after intervention with VR and conventional therapy in inpatients with COVID-19, during hospitalization and after 7 and 30 days of follow-up after hospital discharge.

### Secondary Outcome

We will analyze changes in mood, satisfaction, and enjoyment regarding the interventions, RPE, and functionality in both interventions.

### Statistical Analysis

Statistical analysis will be performed using IBM-SPSS (version 26.0, IBM Corp., Armonk, NY, USA). As dependent variables, all HRV indices will be considered, as well as test scores of Barthel Index, TUG, MRC, BRUMS, ES, VASS, Borg Scale, Sao_2_, RR, and HR. If the data meet the assumptions of normality, multiple analysis of variance will be used to compare the study groups (virtual group and conventional group) and intragroup comparison (virtual and conventional groups, when comparing the same subject), with least significant difference *post-hoc* test. If the data do not meet the assumptions of normality, the differences between the groups will be analyzed using the Kruskal–Wallis test. Dunn *post-hoc* tests will be performed on each pair of groups, with Dunn–Bonferroni post-test on each pair of groups. The same tests will be applied for *p* < 0.05, which will be considered significant.

## Discussion

Rehabilitation was a critical aspect of the healthcare systems during the COVID-19 pandemic; therefore, it is important to prepare new interventions that allow healthcare providers to maximize responses to future rehabilitation challenges (Stein et al., 2020). COVID-19 survivors suffer from reduced lung function, critical illness polyneuropathy and myopathy, cardiorespiratory deconditioning, and impairment in all activities of daily living. Hermann et al. (2020) demonstrated that cardiopulmonary rehabilitation could be performed safely and with beneficial effect to COVID-19 patients, as long as proper safety precautions, close medical management, and supplemental oxygen are available and used if needed.

Moreover, despite studies reporting successful use of the VR in management of different respiratory disabilities (Mazzoleni et al., 2014; Alvarez et al., 2017; Jung et al., 2020; Rutkowski et al., 2020; Taslimipour et al., 2020), lung cancer (Abushakra and Faezipour, 2013), post-thoracotomy lung cancer (Hoffman et al., 2014), and cystic fibrosis (Salonini et al., 2015), the knowledge of the use of this technology in a hospital environment is still scarce. Thus, we organized this study to verify the possibility of using software with VR tasks to encourage physical activity during COVID-19 treatment. Although we hypothesize that all participants will improve in motor, functional, and physiological parameters independent of the intervention (conventional or virtual environment), the use of virtual tasks could provide a more engaging intervention. We can speculate that these results will have a positive influence on rehabilitation programs designed for these patient groups and can be used as an adjunctive for conventional therapy, reducing the suffering of patients during hospitalization. This protocol study therefore aims to answer two important questions:

1. Engagement and motivation: So far, few studies have addressed the motivational and engagement issue within rehabilitation programs (da Silva et al., 2020a). According to Karloh et al. (2020), conventional intervention in rehabilitation following international guidelines does not promote enough motivational changes to ensure engagement and maintenance of physical activity. New technologies (such as platforms and software for rehabilitation) have been suggested as alternatives to improve access and increase capacity of conventional outpatient rehabilitation; however, there has not yet been evidence of a positive impact on behavioral outcomes. Furthermore, an underactive reward system dampens an individual's motivation to engage in activities that are usually experienced as pleasurable. COVID-19 provides the perfect stage for the propagation of demotivation cycle; decreasing engagement, it could also negatively impact mental health (Hagerty and Williams, 2020). Thus, we believe that VR intervention during hospitalization will provide a differentiated form of intervention, positively impacting motivation and engagement in people who are under treatment for COVID-19.
2. Development and use of non-commercial games: A systematic review by Bonnechère et al. (2016) showed that in most cases the introduction of commercial training games for physical rehabilitation offered positive results. However, commercial games are designed for entertainment and are sometimes unsuitable for rehabilitation; there is no possibility of controlling important rehabilitation variables, and it is difficult to adapt the game to the patient necessity (Alankus et al., 2010; Crocetta et al., 2018). An important question is the potential and future use of customized serious games, defined as a game developed for specific target (Leal et al., 2020). Therefore, for the present study, we selected a serious game developed for individuals with physical difficulties to encourage and enhance motivation to engage in physical therapy during hospitalization.

We believe that the results of this study will provide scientific support in the use of VR software for rehabilitation of patients with COVID-19 during their hospitalization.

## Ethics Statement

The studies involving human participants were reviewed and approved by Ethics Committee of the Federal University of São Paulo, under the number CAAE: 33244620.5.0000.5505. The patients/participants provided their written informed consent to participate in this study.

## Author Contributions

TS designed the study, revised the manuscript, performed the statistical analyses, and interpreted the data. PO, JD, AS, CR, and RG collected patient data and drafted the article. ED, MF, ÍM, DS, VB, LA, and HS provided assistance on patient data collection and revised the manuscript. SB and CF revised the manuscript critically for intellectual content. CM coordinated the study, drafted the article, and revised the manuscript critically for intellectual content. All authors read and approved the final manuscript.

## Conflict of Interest

The authors declare that the research was conducted in the absence of any commercial or financial relationships that could be construed as a potential conflict of interest.
